# Machine Learning in Orthopedics: A Literature Review

**DOI:** 10.3389/fbioe.2018.00075

**Published:** 2018-06-27

**Authors:** Federico Cabitza, Angela Locoro, Giuseppe Banfi

**Affiliations:** ^1^Dipartimento di Informatica, Sistemistica e Comunicazione, Universitá degli Studi di Milano-Bicocca, Milan, Italy; ^2^IRCCS Istituto Ortopedico Galeazzi, Milan, Italy

**Keywords:** machine learning, deep learning, orthopedics, literature survey, predictive models

## Abstract

In this paper we present the findings of a systematic literature review covering the articles published in the last two decades in which the authors described the application of a machine learning technique and method to an orthopedic problem or purpose. By searching both in the Scopus and Medline databases, we retrieved, screened and analyzed the content of 70 journal articles, and coded these resources following an iterative method within a Grounded Theory approach. We report the survey findings by outlining the articles' content in terms of the main machine learning techniques mentioned therein, the orthopedic application domains, the source data and the quality of their predictive performance.

## 1. Introduction

This paper is one of the first contributions to systematically address the following question: Will ‘intelligent machine learning’ revolutionize orthopedic practice? This is a question that, although implicit in many recent editorials, was also raised explicitly, although with a narrower scope (Berg, 2017). Our endeavor to address this question does not rely on any oracular capacity or exercise in futuristic imagination. Rather, in this paper, we shed some light on the extent to which computational techniques and methods that can be put under the above-mentioned evocative rubric of “Machine Learning” (ML) have been used thus far and reported in the specialist literature with regard to musculoskeletal problems and related health conditions.

Therefore, in what follows, we will report on a systematic literature review of the journal articles published since 2000, in which authors claim to have used any of the most common ML techniques to address a question in musculoskeletal research. Surveying these applications, classifying them according to a simple framework and reporting on the accuracy and quality achieved by these methods is our contribution to attempt to address the above-mentioned question, if to actually answer it is substantially impossible.

Firstly, it is necessary to circumscribe the scope of our research by supplying a definition from ML. Although any definition covers a broad topic only partially and often from a (professionally) biased perspective, exposing a clear definition of what we intend with ML will help the reader interpret our review choices and findings. We will not arbitrarily forge “yet another definition” of this research field at the intersection of computer science, statistics and data science (Deo, 2015). Rather, we will ground on (and clarify and extend) some of the best contributions to this aim that have been recently proposed in medical literature.

We intend ML as the study of how computer algorithms (i.e., machines) can “learn” complex relationships or patterns from empirical data (Wang and Summers, 2012) and, hence, produce (mathematical) models linking an even large number of covariates to some target variable of interest (Obermeyer and Emanuel, 2016). In the medical field, this means to be able to predict, given a set of radiological images from a Picture Archiving and Communication System (FPACS), lab results from a Laboratory Information System (LIS) or data extracted from Electronic Medical Records (EMR), sensor networks, or specialty electronic registries, for example, a diagnostic label (as in diagnosis), an outcome level (as in prognosis, assessment, and monitoring), an exam value or risk score (as in regression, and prognosis as well), or an identifier of some treatment option (as in therapy), to help physicians make more efficient and accurate decisions.

## 2. ML in a nutshell

The above definition, as broad and plain as it may appear, requires some clarification. Defining ML as a study is a general means to hint that this same label has been attached to a research field, as well as (by metonymy) to both its main object (i.e., how machines can improve their task performance—or “learn”—from data and experience), to different classes of techniques to model the relationship among data variables (such as random forest and neural networks) and even to the single models that ML practitioners build by means of these techniques and applying them to specific data sets. Although this may appear to be an imprecise picture, it reflects the terminological vagueness that characterizes the entire ML discourse. This is likely due to the fact that these techniques have been initially developed by practitioners belonging, as hinted at above, to different communities and have been applied to a huge number of different application domains.

In this vein, we placed the word “learn” in inverted commas. We did this because we deem it counter-productive in fields other than computer science (where metaphors are often used as a note of color to sophisticated mathematical models) to associate automatic procedures of incremental function optimization, which is what ML is all about, with an anthropomorphic element that can suggest something about the inner functioning of ML-based decision support systems^1^. This process, far from being confused as learning *by* something, like a machine, was technically considered a broad family of statistical methods by which a discriminative function could improve its detection accuracy over time, by changing some of its parameters so that a cost score (anyhow defined) becomes minimized as well. With a “discriminative” function we merely intend a mapping between some input, for example, an array of clinical values or of pixels from a medical image (what statisticians would denote as an X, that is an array of possible values in a multidimensional space) to an output value (a *y*), often denoting an element from a set of possible answers: this value can be one between, for example, positive or negative outcome, as in binary classification (which is a kind of discrimination), or one from a set of possible diagnoses—as in multi-class discrimination—in the hope that this latter value is the “right one.”

In ML, different techniques are employed to guess a hypothetical function *f*′, ŷ = *f*′(*x*), which is progressively adjusted to make the difference among *y*, the real intended output and ŷ, the output of the function, as little as possible. The function *f*′ is what is commonly called an ML model. Most of the techniques build and optimize a model *f*′ by giving the *function builder* (an algorithm also called *learner*) lots of pairs 〈*x, y*〉, the so-called *ground truth*, which is a set of input data (*x*) that a certain human has already “labeled,” that is, associated them with their correct *y*; examples of labels are the correct diagnosis for each radiological image, or whether a particular patient (i.e., a specific *x* record) died within three months from the treatment (being *y* either yes or no, 1 or 0).

What we have just outlined is the gist of the so-called *supervised learning*, which has thus far been the most popular ML approach in the medical sciences. As the name may suggest, in the so-called *unsupervised learning*, the correct *y*s are not known or given, and the *learner* infers them on the basis of the extent each input data point (i.e., a single *x* in the above multidimensional space) is close to the others (where proximity is akin to similarity), assuming that the closer the points, the more likely they are to be associated with the same (or similar) *y*. As different values of *y* can be considered as different labels attached to all of the *x* associated with those values by the *learner*, the discrimination produced by an unsupervised algorithm is called *clusterization*.

Delving into the details of the most common model, families would be beyond the scope of this review, as well as of the main challenges entailed by the “learning” process. Good introductory papers to the main ML techniques and the related challenges can also be found in the medical literature, e.g., (Tomar and Agarwal, 2013; Deo, 2015; Madelin et al., 2015; cab, 2017), which this contribution does not want to replicate. In fact, this introductory section was intended to merely make the reader more acquainted with the terms that we will use in reporting the results of our literature review. For this reason, the last distinction we recall is the one between traditional, or as it has been recently called *conventional learning*, and *deep learning* (LeCun et al., 2015).

To simplify certain complex aspects, the main difference between these two ML families of techniques lies in the fact that conventional techniques require a manual (human) intervention in transforming the raw input data (*x*) so that this is suitable for the *learner* to build the mapping function *f*′ efficiently (i.e., to produce what is called a *feature vector*). Since this manual preprocessing, called *feature engineering*, is usually time consuming and requires considerable domain expertise, it has been considered unfeasible for many tasks such as image pattern detection and speech recognition (where the input can comprise of millions of data points) until deep learning techniques had been refined and broadly used. These techniques automate the raw data engineering by automatically finding the optimal inner representation needed for the discriminative (mapping) task. This latter representation is obtained as the final step of a series of intermediate representations resulting from the composition of simple but non-linear modules, which each transforms “the representation at one level (starting with the raw input) into a representation at a higher, slightly more abstract level” (LeCun et al., 2015). Each module can be thought of as a layer, and each subsequent layer can be considered as being “deeper” than the previous ones: in these terms, the typical multi-layer architecture of an artificial neural network is considered to be *deep learning*. Deep learning enables the tackling of huge volumes of data (the so-called *big data*) in an efficient, although often inscrutable, manner. Their efficiency constitutes the big advantage of *deep learning* techniques over conventional ones, while their black-box nature is often considered the main source of concern for their wide application to the medical field (Cabitza et al., 2017b), where explanations for the proposed output must be either explicit or somehow made available to the human decision-maker (Caruana et al., 2015).

## 3. Literature review: method

**Figure 1:**
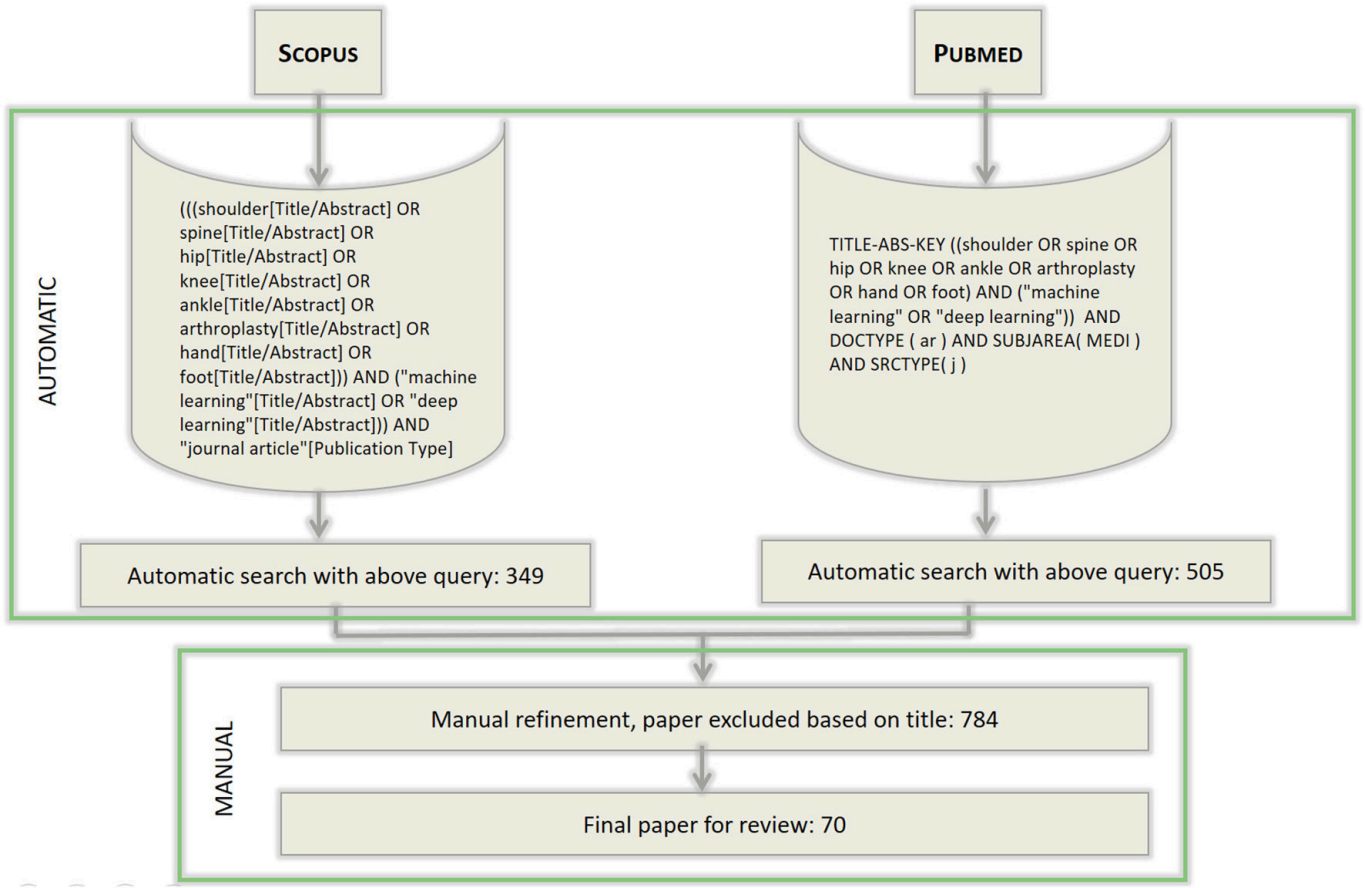
The workflow of search and selection of papers. The syntax of the queries differs for the two search engines used, but the search terms are the same for both the searches. The queries were last executed on November 2017.

The literature review process that we conducted to address the research question reported in section 1 is depicted in Figure 1. To survey the pertinent literature sources, we executed a search on both Medline (through the Pubmed search engine) and Scopus Elsevier databases; the inclusion criteria are reported in the figure. These searches were aimed at retrieving the scientific papers that had been published in the indexed journals and whose authors claimed to have used any ML techniques (by using either the phrase “Machine Learning” or “Deep Learning”) for a major anatomical part of the human musculoskeletal system. To ascertain the potential extent of the components of such a system—which includes the joints, ligaments, muscles, nerves, tendons, and skeleton structures—we focused on six high-level structures: spine, hip, knee, ankle, hand and foot, as well as on a general procedure, that is, arthroplasty. The structural keywords were selected because they are likely mentioned in either the title or the abstract in papers focusing on any of the above anatomical sub-systems or more particular interventions (e.g., spine is likely mentioned in papers on vertebral arthrodesis); on the other hand, arthroplasty was selected as a conveniently general term by which to capture various procedures aimed at restoring joint function after common problems such as arthritis or trauma, without limiting the review to the more common prosthetic joint replacement.

**Figure 2:**
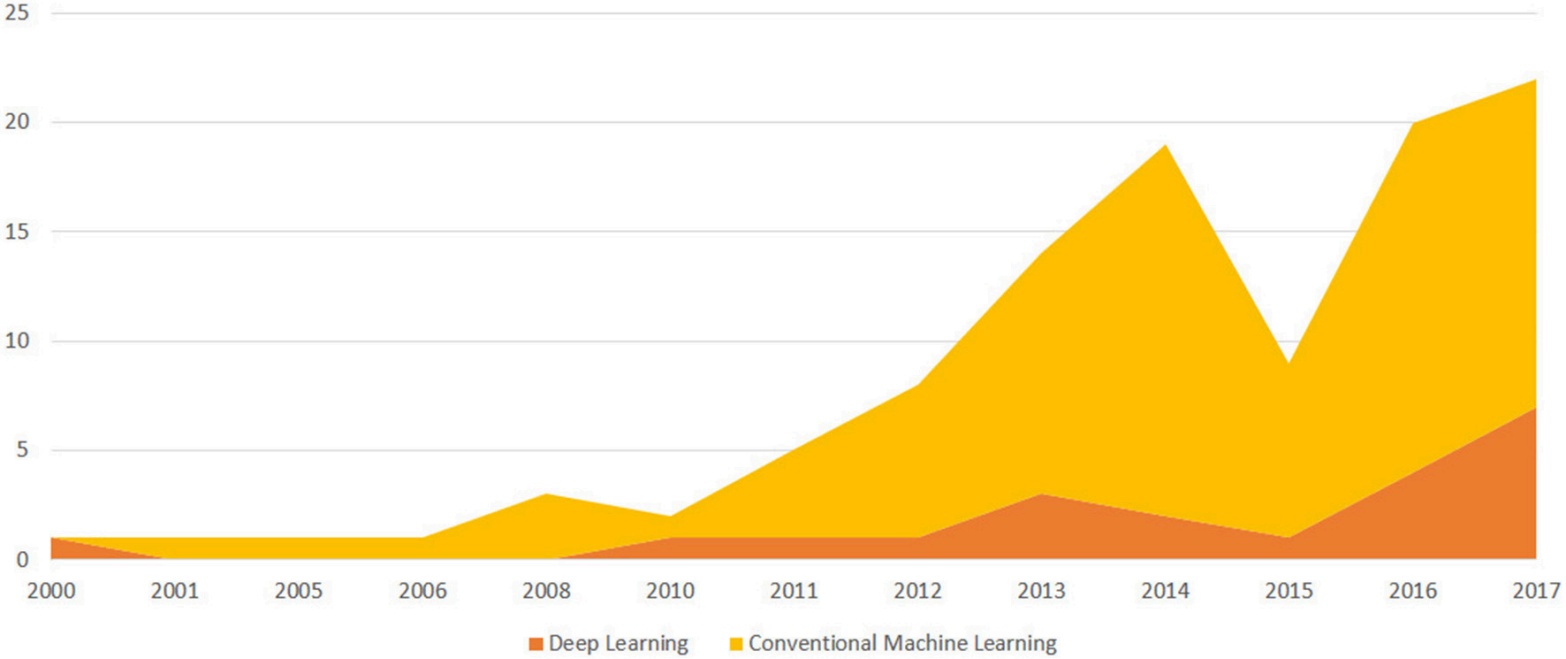
A temporal evolution chart depicting the number of papers published each year since the year 2000 and included in the survey because it discusses an ML application to Orthopedics. We distinguish between papers that discuss deep learning models and conventional ML models.

**Figure 3:**
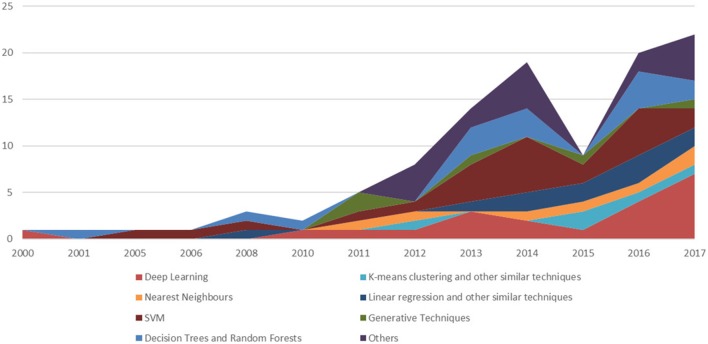
A temporal evolution chart depicting a more detailed picture than the one depicted in Figure 2. For each year, we depict the number of papers stratifying the resulting trends, using the main ML technique.

In doing so, we purposely focused on clinical orthopedics, while also not disregarding surgical procedures and the application of ML techniques to these orthopedic domains. With regard to the techniques surveyed, we are aware that in the past, certain techniques that are nowadays considered to be “ML” (and denoted as such in the specialist articles) once were not denoted as such (but rather considered “statistical learning,” or merely statistical methods without a specific common denotation, as mentioned in section 2). That notwithstanding, in this paper, we purposely focused on this class of applications and on studies in which the authors explicitly claimed to contribute to the growing number of studies that apply ML to orthopedic problems (see Figures 2, 3 for a more detailed classification of ML techniques). Moreover, since the convention of calling the application of a specific technique of ML, that is artificial neural networks (endowed with multiple and hidden layers) in terms of “deep learning” (after the seminal work by Hinton on the so-called Deep Belief Nets Hinton et al., 2006) has been remarkably consolidated in the time span of the review, we also considered this key-phrase explicitly.

A first selection of the resulting papers from the two searches was based on reading their titles and excluding all the irrelevant papers on the basis of this information alone. When deemed necessary, the abstract was also taken into consideration. From a total of approximately 350 papers from the Scopus database, and 500 papers from the Medline one (some of them being present in both the databases), a final selection of 70 papers was considered for a thorough review of their content and coding. The selected papers were divided according to deep and conventional ML techniques and then further into clustering/discriminative and generative techniques.

The content analysis of the papers surveyed relies on the main principles and coding tasks devised within the framework of the Grounded Theory Wolfswinkel et al. (2013). A qualitative analysis software package, NVivo (v.10), was used to assign relevant labels from these 70 articles to sub-themes and to organize the subthemes into main themes. Separate categories for techniques and health problems (i.e., application domains) were constructed. Using an inductive approach, one author defined the initial sub-themes on the basis of the article's content. She then organized these sub-themes into main themes in order to minimize redundancy and overlapping and not to lose important nuances. She associated the content with sub-themes and main themes on the basis of a consensus discussion. When new sub-themes emerged, a re-evaluation of the articles was done to also apply this to the new subthemes. In qualitative research, this iterative process is known as the *constant comparison method* (Wolfswinkel et al., 2013). For each paper, we extracted and systematized the following information: the application domain to which at least one ML model was applied (e.g., vertebrae image segmentation, scoliosis detection, osteoarthritis prediction, gait patterns analysis), and the purpose of the application (i.e., diagnosis, prognosis, therapy or study); the data source exploited in the research (e.g., magnetic resonance images –MRIs, sensors data, patients' data); the applied ML techniques (e.g., decision trees, random forests, support vector machines) and their main objective (e.g., classification, clustering, regression); finally, we also considered the evaluation metrics used to assess and validate the model performance (e.g., accuracy, ROC curve, F-score).

## 4. Literature review: results

In this section, we outline the main works collected through our literature survey using the method described in the previous section. We describe the papers that have been divided according to the ML approach for the sake of reference, beginning from the numerous model families that can be considered under the rubric of conventional (or traditional) ML, and then conclude with the most recent ones that employ deep learning methods (usually a multi-layered artificial neural network).

In what follows, we will not mention explicitly or discuss in full detail each of the 70 papers retrieved and analyzed for this literature review, but only discuss a subset of the most relevant ones. Our choice is mainly motivated by reasons of limited space and by the need to provide a practical summary of the results of our analysis. Our informal criteria for this choice are the availability of the full text of the considered papers, whether these have been published in highly impacted journals in the orthopedics field (or closed ones), and whether the adopted ML approach was adequately discussed in the body of the text (however, as mentioned above, delving into the details of each ML technique and model mentioned in the papers has been considered beyond the scope of this survey). In case of two or more papers dealing with the same problem or approach, we opted to discuss the most recent one(s).

### 4.1. Conventional ML

In this section, we begin reporting on the articles in which the authors described the application of certain conventional ML techniques to an orthopedic problem, aimed at developing the models belonging to the following families: decision trees, random forests, nearest neighbors, linear regression and support vector machines (and others).

#### 4.1.1. Decision trees and random forests

In Kotti et al. (2017), Random Forests (RF) were used both for classifying Osteoarthritis (OA) subjects from healthy ones and to attempt to provide a clinical interpretability of the results through the provision of a continuous regression output that, according to the authors, should better mimic the progressive nature of an OA pathology when compared with a crisp classification output. The authors collected kinematic data from patients which comprised ground reaction forces parameters (GRF), and extracted features for each of the three paths axes –X, Y, and Z. The most informative GRF parameters were computed by summing up the prediction error of the observations that were eliminated along the three paths, so that the final outcome of the ensemble could be appropriately averaged by also considering the error-bias. The sensitivity, specificity, accuracy and F1 score were reported for each group of axis features and for the combination of all three. Cross-validation for the three axes showed a mean accuracy of 72.61 ± 4.24%.

Tree Bagging (TBG)^2^ outperformed the other ML methods that were applied to the work of Madelin et al. (2015). In this paper, OA subjects were classified using sodium MRIs of knee joints transformed into radial 3D acquisitions before and after the application of the fluid suppression technique. Each subject was then described with 12 global features extracted from images regions of interest (ROIs) and relative to mean and standard deviation statistics on the sodium concentration in them. The classification task was tested with 16 different classifiers: Logistic Regression (both linear and quadratic), Linear Discriminant Analysis (LDA), Quadratic Discriminant Analysis (QDA), k-Nearest Neighbors (kNN), Naive Bayes (NB), Neural Networks (NN), Support Vector Machines (SVM), Decision Trees (DT), and Tree Bagging. In Madelin et al. (2015), the performance in terms of accuracy with holdout cross-validation for the Tree Bagging method was lower than < 74%.

A probabilistic boosting tree classifier^3^. This method was applied by Mirzaalian et al. (2013) to find a robust segmentation method to 3D vertebra Computer Tomography (CT) spine images. This segmentation was supported by the information on each vertebra shape extracted by means of statistical shape modeling techniques (SSM) and appropriately surrounding each vertebra with a box using a boundary detector learner. For each test image, the trained algorithm estimated the vertebra shape using a mean shape on extracted features and applying the boundary detector to them. After this operation, point meshes were aligned rigidly to and projected onto the SSM model space for their approximation to the mean shape. The classification step was evaluated with minimum error, which yielded a value of 1.37 ± 0.37 mm.

#### 4.1.2. Nearest neighbors (NN)

In Ashinsky et al. (2015) and Ashinsky et al. (2017), a weighted neighbor distance method was used with a compound hierarchy of algorithms first introduced in Shamir et al. (2008) representing morphology (a technique abbreviated with the acronym WND-CHRM) and applied to the problem of OA detection in MRIs of articular cartilage scans. This tool is an image classifier that extracts features from a training set of images, and weights them by discriminating order (for example, through LDA). The features extracted by WND-CHRM are numerical image content descriptors–such as image textures, pixel statistics, polynomial decomposition and high-contrast features—as well as image transforms and pair-wise combinations of these transforms. In the first of the two studies, this technique was used for binary classification. The authors used the Osteoarthritis Research Society International (OARSI) score (Pritzker et al., 2006) as the final output of a multiple linear least-squares regression model to score each cartilage plug, apart from the previous binary classification. This score was assessed manually by two human experts on the same set of images, and both scores were used as the basis for the linear regression procedure. The best separation task was obtained by extracting features with WND-CHRM from T2-weighted (T2W) measurements (among the different scan measurements taken from the original MRI), with an accuracy of 97%, sensitivity of 94%, specificity of 100%. With regard to the regression task, the best performance was given for the same set of features as above (T2W), with the lowest root-mean square (RMS) value of 1.6.

In the second study, the same authors referred to the Western Ontario and McMaster Universities Arthritis (WOMAC) questionnaire (Bellamy et al., 1988) administered to subjects (OA control and incidence cohorts) in order to estimate the progression of OA in MRI scans taken from the same subjects three years earlier. OA progression was estimated by administering the questionnaire to the patients after three years from their last MRI. The WOMAC questionnaire includes 24 questions related to pain scales, stiffness in performing a few daily activities and evaluation of certain physical functions. The respondents were permitted to use three versions of the questionnaire, one for each rating system: adjectival, numerical or visual. In the WOMAC application case, the accuracy gained was 75%, a lower result when compared with the previous study. Different factors were advocated for this difference: in the first study, the data set was less generic in describing OA subjects and OARSI score provided an objective measurement; in the last study, the ground truth was represented by patient's answers of WOMAC, which were more “subjective” and more difficult to relate to image features scoring.

In terms of gait patterns, recognition for gaits classification into healthy and pathological (Dolatabadi et al., 2017), an instance-based discriminative kNN was compared to a dynamical generative model like the Gaussian Process Latent Variable Model (GPLVM). Gait features acquired from kinetic skeletal tracking were divided into self pace (WSP), dual task (WD), and fast pace (WFP) and were used to train the two predictors by letting them observe single or multiple gait sequences of different kinds. The kNN model assigned a class label to each new sequence with a majority voting mechanism among the kNN in the training set. The GPLVM was trained with healthy data and trained to identify the likelihood that an input sequence belonged to the healthy pattern. The input sequence dimensionality was first reduced (the latent space) by Principal Component Analysis (PCA) and then the joint likelihood of low and high dimensionality space (the observed space) was maximized using a gradient descent technique. The initialization step was obtained by mapping latent space to a Hidden Markov Model (HMM), in which hidden states were latent states and where the Viterbi algorithm (Forney, 1973) was used to find the most likely sequence of hidden states for obtaining the observed space. The likelihood of the observed space given the latent state was then the posterior prediction computed by the model. The kNN model obtained a better F1 score (0.95 vs. 0.87) on WSP features discrimination. The generative model obtained a lower micro-averaged error (0.15) on the WSP features.

#### 4.1.3. Linear regression and other similar techniques

In orthopedics literature, several papers use regression methods to model, for example, knee injuries (e.g, anterior cruciate ligament—ACL and posterior cruciate ligament—PCL, Matić et al., 2016), and articular cartilage degeneration for predicting advancements in OA (Pedoia et al., 2017). In the study by Matić et al. (2016), a gait analysis was performed to obtain a model of useful indicators of knee deficiencies (ACL), by means of movement curves. The main indicators of ACL-deficiency were detected in anterior posterior translations (APT) and internal external rotations (IER). After having tested the statistical significance of the APTs and IERs to discriminate between healthy and pathological subjects by means of a Wald test, a binary logistic regression function was modeled to classify the two APT- and IER-independent factors. The decision boundary traced by the regression function was manually assessed by an expert, thereby leading to a successful discrimination rate of two cases out of the three that were manually analyzed. There were no other evaluation criteria in the study, with the exception of statistically significant APT and IER ordered pairs in showing a relation with ACL-deficiency. Higher ATP values revealed that the presence of ACL-deficiency is 1.1758 times more frequent than in lower ATP values (95% CI). Higher values of IER show that an ACL-deficiency is 2.2516 times more frequent than lower IER values (95% CI).

A regression model was also applied in Pedoia et al. (2017) to heterogeneous data, that is, patients, biomechanical and MRI. These data were combined into a Topological Data Analysis (TDA) integration and visualization framework, in order to deal with the problem of supporting the analysis of progressive cartilage lesion in knee OA. Each participating subject was represented as a point in the TDA space and similar individuals were grouped into nodes. In this manner, it was possible to detect population subnetworks, in which subgroups of OA patients could be analyzed in their “syndromic spaces.” The TDA was also tested for its capacity to generate hypotheses and for modeling selective configurations obtained by filtering out different kinds of variables. The logistic regression classification showed an Area Under the ROC curve^4^ of 91.1%, sensitivity of 86.8%, and specificity of 86.8%.

#### 4.1.4. SVM

SVM are used in orthopedics research for disparate kinds of classification tasks and on different data sources. For example, for OA prediction, they are applied on images (Zhang et al., 2013; Nagarajan et al., 2014; Madelin et al., 2015), biochemical (Ahmed et al., 2016) and biomechanical measurements (Yoo et al., 2013a; Phinyomark et al., 2016). In Phinyomark et al. (2016), 100 knee OA subjects and 43 healthy subjects (both equally distributed between men and women) participated in a study where their kinematic behavior was observed, registered and measured. Each gait pattern was represented by angles of interest for each joint and plane of motion. A feature vector representing each subject (i.e., OA and control groups) was then extracted, and a principal component analysis (PCA) was applied in order to reduce the vector dimensionality to a low resolution space that maximized the variability of the original data and created a Principal Component (PC) coefficient matrix for each group. Both the original data and the PC data were analyzed using a conventional statistical method, that is, ANOVA, and by a SVM classifier. A statistically significant difference was found in OA women kinematics with respect to OA men, and the same difference was obtained for healthy subjects from transformed data. This difference affected healthy females who exhibited significantly greater probability of musculoskeletal injuries, as confirmed by previous studies mentioned in the present study. SVM accuracy was assessed to be 99% in correctly separating OA women from OA men, using a ten-fold cross-validation. For healthy subjects, the SVM accuracy was assessed to be 86%.

A similar study is the one by Laroche et al. (2014), where kinematics trajectories were measured for each subject and compared to the WOMAC standard for assessing their reliability in representing the OA severity in affected patients. The training features were extracted by a three-dimensional computerized movement analysis device in which kinematics trajectories were related to the three frontal, sagittal and transverse planes dimensions. The resulting feature vector contained spatial angles, trunk motion and pelvis motion in the three planes for each participant. An SVM classifier was trained to learn whether a gait cycle belonged to a patient or a control subject, and this task reached a maximum accuracy of 90%; the same classifier was used to decide whether a gait cycle represented a significant one for better discrimination, and all the detected trajectories provided discrimination with mean accuracies higher than 70%; and, finally, the classifier was used to decide whether a correlation existed between a maximally discriminating trajectory and the related WOMAC score: this task achieved a correlation of 0.74, with p-value < 0.05.

A third study Nagarajan et al. (2014) used phase-contrast X-Ray CT images to detect geometric features that best characterized condrocyte structures in OA, healthy patients ROIs as well as gray-level statistical features from manually annotated ROIs of the same set of images. For the first set of geometric features, a scaling index method (SIM) was exploited for points distributions analysis, and a statistical analysis was adopted for gray-level patterns. The accuracy of SVM trained with condrocyte patterns reached 95%, while that of the SVM trained with other statistical patterns reached 93%.

The research of Zhang et al. (2013) focused on the automatic knee cartilage segmentation in MR images by using multi-contrast mechanisms of image acquisition, such as for example, T1-weighted FS SPGR, T2/T1-weighted FIESTA, T2/T1-weighted IDEAL GRE water and fat imaging. These acquisitions were used for selecting voxel features based on normalized intensity values, local image structure-based features, and geometrical features. The classifier exploited in this research is a combination of two different approaches, to compensate the limitations of both: SVM and Decision Random Forests (DRF). A sum-product loopy belief propagation (LBP) inference algorithm was used for labeling optimization. In brief, LBP computed the marginal distribution for each unobserved node (voxel), conditional on the observed ones on the general graphical model, and repeatedly applied the belief propagation updates to the subsequent steps. The average sensitivity (0.867) and specificity (0.997) were used to evaluate this approach on different sets of feature vectors.

A further aspect treated with ML was related to knee injuries (Stajduhar et al., 2017). In this paper, sagittal plane magnetic resonance volumes of human knees were scanned and manually labeled for ACL. These ACL were not partially and completely injured according to written diagnoses. ROIs where then selected from images for voxel descriptors extraction. Object recognition techniques such as histogram of oriented gradients (HOG) and scene spatial envelop description (a gist descriptor) were used to reduce the number of potentially representative features for each ROI. Both techniques divided the ROI into their gradient descriptions and retained patches summary information such as scale and orientation. For the classification task, two approaches were applied: SVM and RF. SVM with HOG features outperformed RF, with a ROC of 0.894 and 0.943 for partial and complete ACL rupture detection, respectively.

Another problem where SVM was applied, apart from deep learning approaches seen in section 4.2, is needle entry site localization Yu et al. (2015). Template matching and midline detection methods were applied to ultrasound spine images in order to extract the best classification features. Over 1,000 images were analyzed and accuracy rates of 95% on the training set and 92% on the test set were obtained. The same SVM was then used to detect the interspinous region for needle insertion in approximately 50 video images, and was successful in 45 cases out of 46.

A variant of SVM, namely Least Squares SVM (LS-SVM) was used by Adankon et al. (2012) to discern among scoliosis curve types from 3D trunk images acquired by means of optical digitizers. Once acquired, the 3D back images were divided into horizontal slices, and each slice was decomposed into patches. Thoracic and lumbar geometric descriptors were extracted and, after a proper reduction transformation with PCA, they were used as classification features. LS-SVM differs from SVM in the formulation of error minimization, which corresponds to a regression problem (least squares data fitting). In this manner, there is a simplification in the parameters of the SVM, which makes the solution become a linear system instead of a quadratic problem. Furthermore, two hyperparameters were selected in the study instead of one in order to regularize the trade-off between error minimization and margin maximization of both positive and negative examples. A Gaussian kernel with different weights for toracic and lumbar features were considered as the function space. For 165 samples, the approach yielded an average success rate of 95%. The lowest performance of the system was produced during the detection of the double major curve vs. the thoracic major curve, which were often confused. A similar study Ramirez et al. (2008) attempted to detect curve changes in scoliosis by applying different ML techniques: SVM, DT and a logistic regression classifier. SVM yielded the best outcome on 141 spine radiographs, with an accuracy of 86%.

A Computer Aided Diagnosis (CAD) system to automate the problem of lumbar inter-vertebral discs (IDV) degeneration diagnosis was implemented by Oktay et al. (2014). This approach was based on the analysis and feature extraction from both T1-weighted and T2-weighted MR images, in order to obtain different types of features: intensity, texture, planar shape, and context. Each of them identified a relevant aspect of IVD pathologies: intensity revealed water loss of herniation by means of high-contrast representations; the planar shape was supposed to indicate disc degeneration; the context was registered for comparisons reason; texture data were supposed to reveal important information regarding anomlaies of discs aspects. The SVM applied to automatically detect IVD pathologies was evaluated with an accuracy of 92.8%, a sensitivity of 94.6% and a specificity of 89.8%.

An analogous LS-SVM was used by Silver et al. (2006) for a double-fold purpose: assessing shoulder strength for healthy vs. pathological classification and ascribe a shoulder strength score to rotator cuff pathology patients in post-operative shoulder function. The data collected for the first experiment were measurements of shoulder strength (isometric measures) at different points in time: pre-operative, 6 months and 12 months post-operative. The results yielded an ROC curve of 0.87. For the second experiment, a different LS-SVM was trained with pre-operative and shoulder data of uninvolved patients. A geometric distance was then computed between these data and those of involved patients. A statistically significant test showed that the SVM was only able to discriminate between the post-operative involved patients, (*p*-value < 0.004) and between the uninvolved and involved pre-operative patients (*p*-value < 0.001).

#### 4.1.5. K-means clustering and other similar techniques

Apart from the research by Thong et al. (2016), already described in section 4.2, another orthopedic research used clustering techniques on image data (Kruse et al., 2017b). In the above study, over 10,000 patients were divided into clusters representing different fracture risks levels: high, average and low. The features were extracted from DXA scans (e.g., lumbar and hip region Bone Mineral Density—BMD), and other clinical information, such as primary care visits and co-morbidity in women subjects with the Charlson Comorbidity Index (CCI). Clustering was formed by standardizing variable means using Euclidean Distance, and were optimized by the Ward's method to minimize intra-cluster variance. Other descriptive statistics were computed on the data for unstandardized variables and on the identified clusters. The hierarchical agglomerative clustering (HAC) procedure computed nine clusters, where the following were the most discriminating features: the BDM, with high fracture risk associated with very low BDM, average fracture risk associated with average DBM, and low fracture risk associated with high to very high BDM; age and specific medications (for example, the use of antiresorptive treatments in peri-menopausal women and medical treatments for previous fractures in other subjects); and CCI. The study lacks a quality evaluation metric.

In a third study of our review, the problem of locating ACL and PCL in T1-weighted MRI scans was tackled by applying a variance of the fuzzy C-means method (Zarychta, 2015). This method measured the fuzziness entropy, which highlighted the difference in intensity of neighboring pixels of an image. The segmentations of ACL and PCL were determined by the fuzzy connectedness method, measuring the weakest link strength in a pixels path of a fuzzy-affinity graph, in order to augment the segmentation power. A feature vector of expressive measurements of healthy and pathological ACL and PCL was extracted and assessed by two expert radiologists. The features extracted included surface and perimeter of the ACL and PCL areas, ratio between the extracted area and the ROI area, the A-length (maximum start and end distance of the skeleton) and the B-length (maximum distance between the top and bottom skeleton edges), and the A-length/B-length ratio. The same radiologists stated that in 89% of cases, both ACL and PCL segmentation and features extraction was correctly defined.

The study of Dam et al. (2015) used a freely available knee image data set resulting from a challenge initiative called “Segmentation of Knee Images 2010” (SKI10), MRIs from the OA initiative (OAI), and MRIs from the Center for Clinical and Basic Research (CCBR) to perform automatic bone and cartilage segmentation from MRIs for OA detection. In this study, a kNN technique was exploited on an ROI-based voxel classification and a set of structure-wise classifiers for feature extraction to obtain a classification strength for each voxel and map this strength to a class label. The performance was measured with accuracy given by the Dice volume overlap, with the highest result of 86.6% on an OAI validation set.

#### 4.1.6. Other discriminative techniques

##### 4.1.6.1. Gradient boost machines (GBM)

Gradient Boost Machines (GBM) were used in two studies Atkinson et al. (2012); Kruse et al. (2017b) for automatic bones fracture prediction in subjects at risk, such as menopausal women. In Atkinson et al. (2012), the novel idea was to collect features other than the standard areal bone mineral density (aBMD) to find other strong predictors or a combination of them that are able to improve the fracture prediction outcome. Three sets of variables were then extracted from quantitative computer tomography (QCT) images of both distal forearm fractures (DFF) and vertebral fractures (VF). The same data were collected for two random control groups. These were variables that pertaining to the following aspects: bones density (aBMD), derived by dual-energy X-Ray absorptiometry (DXA); bones geometry, derived by spiral QCT extractions of volumetric bone mineral density (vBMD) of cortical and trabecular bones; and bones micro-structures, derived by high-res peripheral QCT imaging (HRpQCT). These features were used in different combinations to feed the GBM and logistic regression models that were adjusted and evaluated based on ROC results on each combination of variables. In order to prevent overfitting^5^, DFF GBM were also used to predict VF response variables and vice versa. The results suggested that using a model with the entire set of variables (267) yielded an ROC of 1.0. Other ROCs were computed for the following combinations: DXA 0.95, HRpQCT 0.96 and spital QCT 1.0.

The study by Kruse et al. (2017b) aimed at assessing hip fracture prediction by using DXA data and by training 24 different learners. Among these, the final choice was to adopt a bootstrap aggregated (of) flexible discriminant analysis (bagFDA) model and an extreme Gradient Boosting (xgbTree) model, based on decision trees. Several predictors were computed and assembled for the two approaches. The combination of both (11 variables for women and 9 variables for men) aimed at achieving a trade-off between discriminability power (evaluated in terms of ROC) and best calibration of probabilities (a technique to retain the output of a discrimination result also as a probability prediction of that result, such that the probability value can be interpreted as belonging to a 95% confidence interval–CI). The results for the women cohort were opposite to those for the men cohort: for the first group, the bagFDA approach yielded the best choice result, with ROC 0.91 (sensitivity 88% and specificity 81%) and the best balance with calibration of probability; for the second group, the xgbTree result was deemed the best performing approach, with ROC 0.89 (sensitivity 100% and specificity 69%) and the best balance between discrimination and calibration of probabilities.

Other ensemble methods were applied to the problem of skeletal maturity assessment (Cunha et al., 2014). Bootstrap aggregating (bagging) was used to obtain an aggregated predictor, beginning from using bootstrap replicates of the training sets as multiple predictors. The stacking technique served to identify the meta-learner that maximized the reliability of multiple predictors. A total set of 338 features of hand bone ROIs was the input to several regression schemes. Linear regression and rule-based regression (M5P) results were averaged by means of bagging and evaluated with mean absolute error (MAE), yileding a result of 10.16 for M5P. The result suggested that ensemble schemes could rely on a less dependent scheme with respect to a single regression method and that using them on each ROI may improve the results at a small computational cost.

##### 4.1.6.2. LDA and other similar techniques

LDA was used in orthopedics for automating the diagnosis of OA and the prognosis of cartilage loss risk (Marques et al., 2013). The authors analyzed MRI scans of both knees, taken before and after two years, of approximately 150 subjects aged between 30 and 72 years of age. The same scans were manually assessed by an expert radiologists who scored them using Kellgren Lawrence (KL) grades (Kellgren and Lawrence, 1957). The medial tibial cartilage volume was estimated in both the sets of images, after a segmentation procedure and the extraction of the ROIs representing the trabecular bone structure. The extracted features were used to train an LDA classifier able to select the best combination of them for relating the bone structure markers to the prediction of cartilage volume loss. The results of the ML output were evaluated with an ROC of 0.92 for correct classification of healthy vs OA affected ones, and with odds ratio (OR) for the prediction of cartilage loss volume by quantifying bone structure. It was shown that the vertical trabecularization was the most related to cartilage loss progression (with an OR of 16).

A similar study was conducted earlier by the same authors (Marques et al., 2012) by using six different ML classifiers for analyzing trabecular bone structure and quantifying OA risks. The best approaches were LDA (witn a ROC of 0.82) and the weighted k-NN (with a ROC of 0.81). The same approach was one of the best performing ones in the task of skeletal maturity assessment among over 20 ML methods that were tested in the same study (Cunha et al., 2014, see section 4.1.6.1 for details on this research).

A further study aimed at assessing the validity of LDA, QDA, and SVM in modeling hand motor impairments for assessing cervical spine disorder (Lee et al., 2016). Pre-surginal patients underwent a test with a handgrip device, which was able to track two waveforms: sine waveform, to measure hand muscle movements, and step waveform to measure handgrip force. Other features were clinical data such as age and weight and index of perceived hand impairment. With these features as input, the QDA outperformed the SVM and LDA approaches, with a c-statistic of 0.89, a true positive rate of 0.83 and a true negative rate of 0.87.

#### 4.1.7. Generative techniques

In orthopedics research, a generative model^6^ was applied to the prediction of the progression and shape of idiopathic scoliosis affected spines, beginning from a 3D spine reconstruction of X-Ray images (Kadoury et al., 2017). The authors modeled a probabilistic manifold structure, a geometric space with appropriate structures and transform operators, where high-dimensional data were reduced in dimension while preserving high-dimensional properties. The manifold structure, in the reduced dimensionality, best discriminated the curves of spines between progressive and non-progressive by maximizing the distance between these two conditions. This was obtained through a discriminant graph-embedding approach able to maintain the similarities between high-dimensional points in the low dimensional space, such that the mapping operation between the two spaces was locally linear and preserved the distance between neighbor points (a result which was feasible owing to the Riemannian manifold properties of the model space). One within-class similarity graph and one between-class similarity graph were modeled to maximally separate features in the manifold space. Another component of the model was a prior probability which was used during the linear mapping between high-res and low-res spaces in order to compute the distribution of each specific mapping. To predict the deformity progression of a 3D spine baseline reconstruction, this reconstruction was projected onto the manifold space to identify the neighborhood samples. A geodesic curve was used to represent the spatio-temporal evolution of the curve by means of regression methods, which aimed at estimating the progressive deformities of the curve. This was obtained by applying the inverse transformation operation, that is, from a given point on the regressed curve to the high-dimensional space, until a predicted 3D representation of the output spine was obtained. The approach outperformed other ML approaches, both discriminative (SVM) and generative—Locally Linear Embedding(LLE) and the Locally Linear Latent Variable Model (LL-LVM)—with 81% accuracy, 87.9% sensitivity, 75% specificity and 0.85 ROC.

### 4.2. Deep learning

In Thong et al. (2016), both deep learning and clustering techniques were applied to the automatic detection of Adolescent Idiopathic Scholiosis (AIS). An artificial neural network with a stack of auto-encoders architecture was trained to optimize the encoding-decoding of 3D spine model vectors, reconstructed from a cohort of 915 biplanar radiographic images. After the encoding of the most salient landmarks for AIS Lenke types, decoding vectors represented the lowest-resolution set of features with the most complete variations of morphological information for AIS classification. An iterative optimization technique applied to the neural network aimed to minimize the difference between the encoded input vectors and the decoded output vectors. In a second step, an unsupervised clustering method based on k-means was applied to the 3D spine representations vectors obtained in the preceding phase, and eleven clusters were formed and validated against standard geometric 3D spine indices (e.g., Cobb angle, kyphotic and lumbar lordosis angles, pelvic incidence, etc.), and tested for significant differences either by ANOVA statistical test or by a manual clinical assessment of the clusters centroids by an expert surgeon. The main factors helping the algorithm identify AIS clusters were the main thoracic and thoraco-lumbar deformities (MT, TL) of Lenke types 5–6 features, apical vertebra locations features and the single, double and triple major curves characterizing Lenke types 1–4 features.

The authors of Jamaludin et al. (2017) applied an ML method to MRI vertebral images to automate the grading of lumbar IVD status in patients' back pain diagnosis. T2 sagittal MRIs were input into an image analysis algorithm based on the image analysis technique called “part-based models for objects detection,” which labeled T12 to S1 vertebral bodies. These images were annotated beforehand by an expert spinal radiologist with eight radiological scores for each IVD (the average total of scored IVDs per patient was six discs). A convolutional neural network (CNN) was then trained to learn radiological features and predict grading, beginning from the previous annotated discs and the manual scores, for a total of six features extracted and used to predict the output grading. Each feature was graded by a binary present/absent grade or by a multi-class grade, depending on the characteristic of the pathology: Pfirrmann Grading and Disc Narrowing were assigned a multi-point scale output, whereas spondylolisthesis, central canal stenosis, endplates defects and marrow signal variations were predicted as being present or absent. The approach was evaluated by using four different measures: class-average intra-rater reliability of the expert radiologist, accuracy between the automatic process and the radiologist, intra-rater reliability coefficient of the expert radiologist and between the system and the radiologist. The best accuracy rate (95%) was obtained for the prediction of spondyolisthesis.

In Forsberg et al. (2017), a similar approach based on clinical annotated MRIs with information labels for each spine vertebra was used to train two deep learning pipelines, one for cervical and one for lumbar vertebra detection and labeling. For each pipeline, two CNNs were used to detect more general and more specific vertebra features, respectively. Further, a parts-based graphical model based on a layered graph was constructed to filter out false positives and label each vertebrae correctly. In this graph, each layer represented the detected vertebrae and previously seen configurations of detected and labeled vertebra, and a distance function based on mean and covariance adjacency matrices measured the shortest path between them. The highest performance of this deep learning approach showed a sensitivity of 99.8, a specificity of 100 and an accuracy of 99.8% for the detection task and 97% for the labeling task.

A deep learning approach was also exploited by Olczak et al. (2017) for the automatic detection of fractures. In this study, several freely available multilayer neural networks were trained to identify fractures, body parts, exam view, and laterality (either right or left) in X-ray images. Toward this aim, over 250,000 ankle, wrist, and hand radiographies were analyzed. While the fracture diagnosis could only be extracted from written reports attached to X-rays, the labels for laterality, exam view type and body part were easily obtained from images metadata. Each of the trained networks underwent several runs, where each image was subject to a stochastic gradient descent algorithm in order to know and discern among the visual and metadata features by minimizing classification errors. Thereafter, the best performing network, a 16-layer Visual Geometry Group (VGG) CNN, was manually inspected for errors by examining the first 400 images of the test set, which were simultaneously analyzed for fractures detection by two expert orthopedics. A gold standard was also used in this phase on the 400 images as a benchmark for measuring the network performance. The best accuracy on the fracture identification challenge was 83%. Secondary outcomes were those obtained on body part (99%), exam view type (95%) and laterality (90%). An inter-observer reliability score was computed for fractures with Cohen's exact Kappa between both the observers and between the observers and the best performing network. The inter-observers score reached 90%, whereas the reliability index between observer 2 and the best performing network reached 86%.

In a similar study, Al-Helo et al. (2013), the authors attempted to learn lumbar wedge fracture diagnoses from CT image labeling, for segmentation and prediction, by applying either a neural network and a k-means approach. The neural net was assessed to attain an accuracy of 93.2% on average for lumbar fractures detection, while the clustering method attained an accuracy of 98% on average, thereby showing a sensitivity of over 99% and a specificity of 87.5%.

Two recent studies, namely Pesteie et al. (2015) and Hetherington et al. (2017), exploited artificial neural networks trained with ultrasound images to automatically detect the optimal vertebra level and injection plane for percutaneous spinal needle injections. They used different ML techniques on the same type of medical images. In the first study, spinal ultrasound images were recursively partitioned into a multi-scale patch sequence, where an Hadamard Transform (Beauchamp, 1984) was applied to each scale level image patch. Hadamard coefficients were used to map typical ultrasound wave-like signatures into region-orientation correlations signatures, to detect discriminating features for different spinal patterns. An artificial neural network was trained to implement this recursive partitioning and to classify each input image as either belonging to or not belonging to the target plane for both epidural and facet joint injections. The results showed the highest accuracy of 95%, a maximum sensitivity of 96%, a maximum specificity of 97% and a maximum precision of 97%.

The second study reported a real-time scanner system (SLIDE) implementing a pre-trained CNN and a finite-state transducer that automatically detected the patient's vertebral level for the optimal lumbar puncture point (Hetherington et al., 2017). The CNN was trained using a transfer learning approach, where an inter-domain knowledge transfer was used as a prerequisite for accurate prediction. On this basis, the CNN was able to recognize three main sites along the patient's vertebral scanning–sacrum, inter-vertebral gaps, and vertebral bones–and was also able to assign probabilities to each of them. A state machine applied a threshold to each probability and triggered a transition to a new state based on the recognition in the previous state of one of the three kinds of scannable body parts. The accuracy obtained in this experiment was 90.8%.

A VGG 16-layers CNN was exploited in hip OA diagnosis (Xue et al., 2017). Over 400 hip X-ray images were first classified into normal and affected by OA by two physicians with over 20 years of experience, and this was considered the baseline of the study to compare the ML tool with. The CNN was then trained with around 45 images and the parameters were optimized after a five-cross validation procedure, obtaining the best accuracy of 87.5%. An ROC curve of 0.94 measured the discriminating performance of the CNN. For the test set, only specificity and sensitivity were given (90.7 and 95%, respectively), and a Diagnosis Agreement Rate (DAR) was assessed among the automatic classification of the test set, the initial expert raters and three final raters of the test set, with clinical experience of 3, 10, and 15 years, respectively. The DAR between the CNN and the initial expert raters was 92.8%. DAR between the initial experts and the more experienced expert was 96.4%. The DAR between CNN and the less experienced physician was shown to be lower than that between the CNN and the more experienced physician (74.7 and 92.8%, respectively).

Several experiments were conducted to compare existing and new deep network architectures in the field of skeletal bone age assessment (Giordano et al., 2016). The proposed methodology of this study was to use a pre-trained CNN (not necessarily instructed with domain images) for feature extraction and to attach to it a single or two fully-connected layers regression network for age estimate, which is fine-tuned with domain images parameters based on the domain-free configuration of the features extracted from the pre-trained CNN. This first experiment aimed at suggesting two main aspects: that the use of off-the-shelf CNN for reducing the training effort (by re-using “low-level kernels” and by customizing only “high-level kernels”) does not affect the results; that the regression layers (tested with four progressive neuronal size) suffices the minimum number of neurons (128) required to perform accurately. In the first part of the experiment, the authors exploited three available CNN: OverFeat, GoogLeNet, and Oxford Net. A second experiment was the settlement from scratch of a new deep neural network architecture: BoNet. Among the different combinations experimented on (number of convolutional layers, number of feature maps for each layer, presence of the deformation layer), the most effective one finally comprised five convolutional layers, such that the last was a deformation layer for managing affine warping; this was followed by a unique 2048-neuron fully-connected regression layer with a final output neuron for age classification. The performance measure of the different experiments, with or without division of the X-Ray images into age, sex and race groups was estimated by MAE between two manual readings of each image and the automatic approach. The performance of the different methods were also assessed against the “Digital Hand Atlas Database System” benchmark. For the first experiment, GoogLeNet was the best performer (with MAE between 0.79 and 1.16). Further, BoNet performed better than GoogLeNet (average MAE of 0.79, against average MAE of GoogLeNet 0.82 and OxfordNet 0.83). A final observation of this study highlighted the difference in the features extracted for bone age assessment between the manual Tanner-Whitehouse procedure compared with the ones extracted by deep network models.

A very recent progress of a CNN based approach for multi-class labeling classification was reported in the study of Liu et al. (2018). The SegNet technology is based on a convolutional encoder-decoder network (CED) and on a 3D simplex deformable model, able to provide high accuracy and efficiency in semantic segmentation problems. This was applied to a freely available knee image data set (the one available from the SKI10 initiative introduced in section 4.1.5) for bone and cartilage segmentation and labeling; the performance accuracy reported was 75.3% for femoral cartilage labeling and 78.1% for patellar cartilage labeling.

### 4.3. Visually supported discussion of the results

In order to present the results of our literature survey visually as well, we provide seven data visualizations, which we describe in the following account. Their description is also intended as a means to discuss the results of the survey and wrap up our reflections.

Figures 2, 3 have been already commented upon and have a simple interpretation: the number of articles on the impacted orthopedic literature that explicitly mention ML (either conventional or deep) has constantly increased in the last 20 years, and approximately tenfold since 2010. As hinted above, this does not necessarily imply that before 2000 there were no ML techniques that were applied to orthopedic problems; rather, this is a sign that these techniques are now increasingly frequently recognized under this clear-cut umbrella term, and ML is becoming an increasingly common topic among orthopedic practitioners and researchers we well.

**Figure 4:**
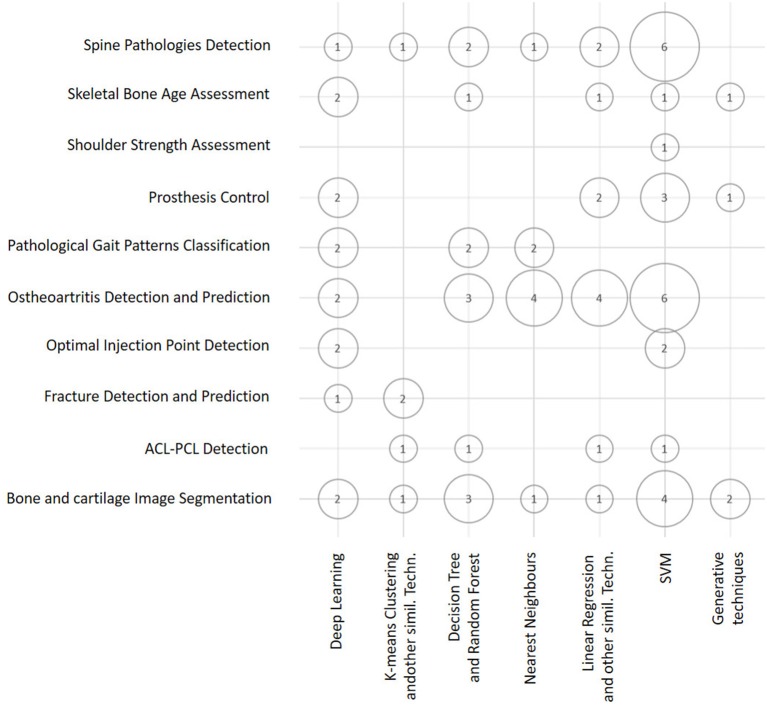
Bubble chart showing the number of papers considered in this survey arranged according to the ML techniques discussed in this section and the main classes of orthopedic application. Papers may have been counted more than once in this chart, depending on all of the ML techniques and applications reported in each of them.

Figure 4 is a bubble chart that presents an overview of the number of publications (proportional to the bubble areas) divided by application domain and ML technique. This chart outlines how the number of studies that employ some kind of ML technique varies for different application domains. By combining these three dimensions, it becomes evident that the largest number of papers (corresponding to the largest bubbles) falls in the areas of Osteoarthritis Detection and Prediction, Bone and Cartilage Image Segmentation, and Spine Pathologies Detection. Similarly, it is evident that diagnostic and prognostic purposes do not differ significantly in terms of the number of papers that deal with them.

**Figure 5:**
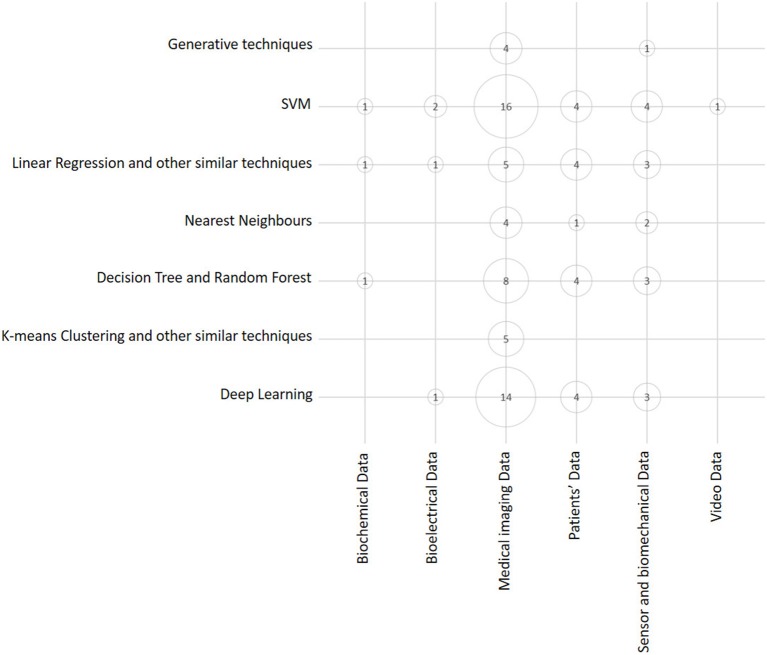
Bubble chart showing the number of papers considered in this survey arranged according to data source typology and main ML class of techniques. Papers may have been counted more than once, depending on all the ML techniques and data sources exploited in each of them.

A similar visual overview is depicted in Figure 5, where we represent the number of articles in terms of ML technique and the kind of source data exploited. This bubble chart shows that both SVM and Deep Learning techniques are the techniques that are most frequently used, and that Medical Imaging Data are the kind of data that are exploited more frequently. This is no surprise, as it may reflect the relatively widespread diffusion of advanced PACS platforms in orthopedic facilities in comparison to EMR and other computer support to clinical (orthopedic) practice (Abraham et al., 2011).

Considering the dimension (axis) that the two above charts have in common (see Figures 4, 5), that is, the class of ML model (or technique), it is evident that the predominant conventional techniques employed in the surveyed studies have been the SVM (Ben-Hur et al., 2008) and the DRF (Boulesteix et al., 2012). In addition, in this case, this result confirms recent technique-oriented surveys, like (Fernández-Delgado et al., 2014), in which, after comparing hundreds of methods and their variants on real-world data, the two above-mentioned models have been associated with the most accurate performances.

Then, for each paper surveyed, in Figure 8 we report (in chronological order and tabular form) the following aspects: the author and year of publication, the most accurate ML model trained in that study, and the kind of data that were used as a source for the training and validation. From this figure, the trends observed above with regard to the application of ML to the orthopedic field become evident: Deep Learning and SVM prevail as main techniques and Medical Imaging prevails as the main data source.

**Figure 6:**
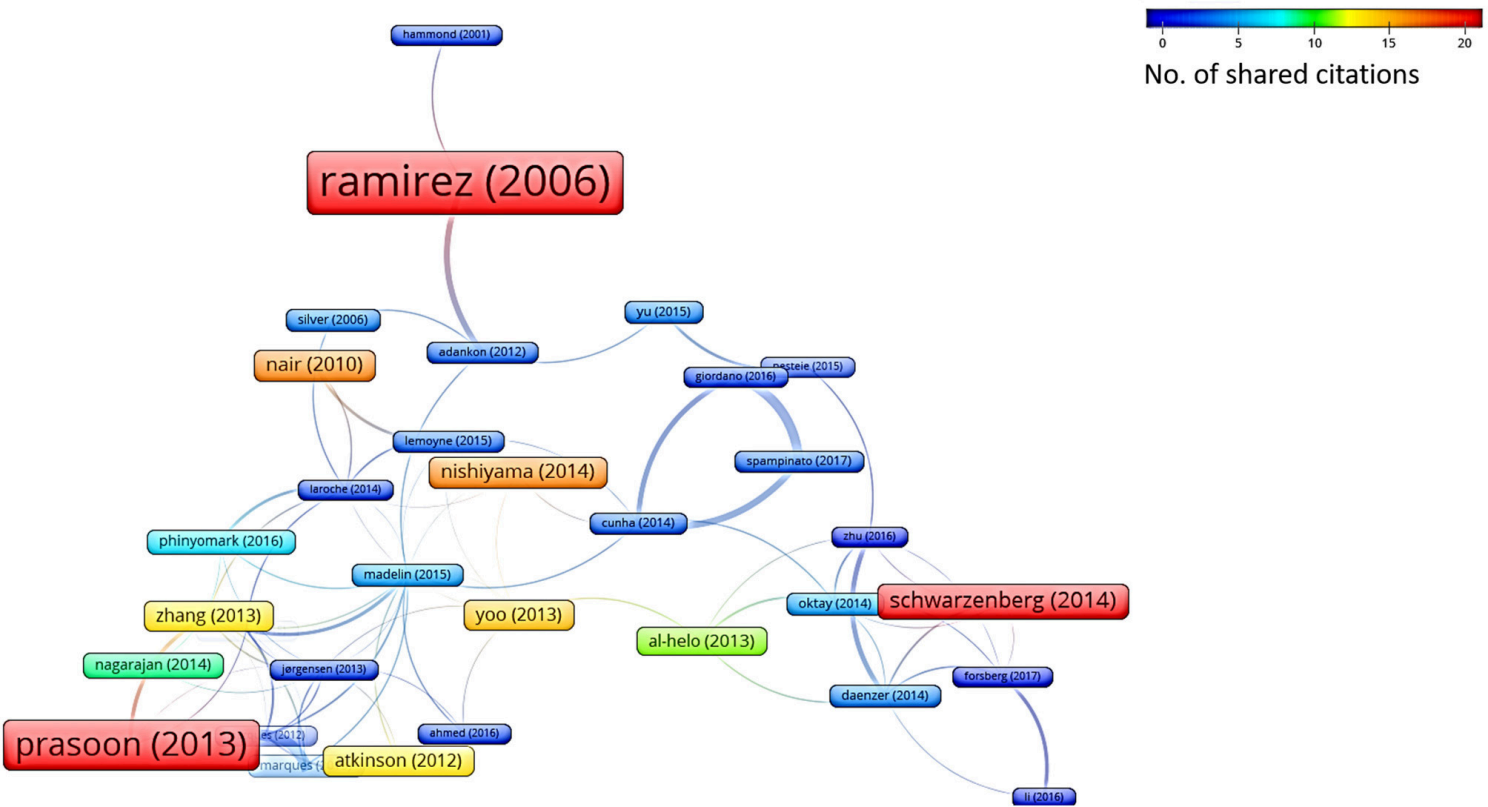
Bibliographic coupling analysis (performed by VOSviewer http://www.vosviewer.com/). The size and color of the nodes represent the number of references that are shared among the papers analyzed. The strength of a link indicates the number of cited references that two publications have in common.

**Figure 7:**
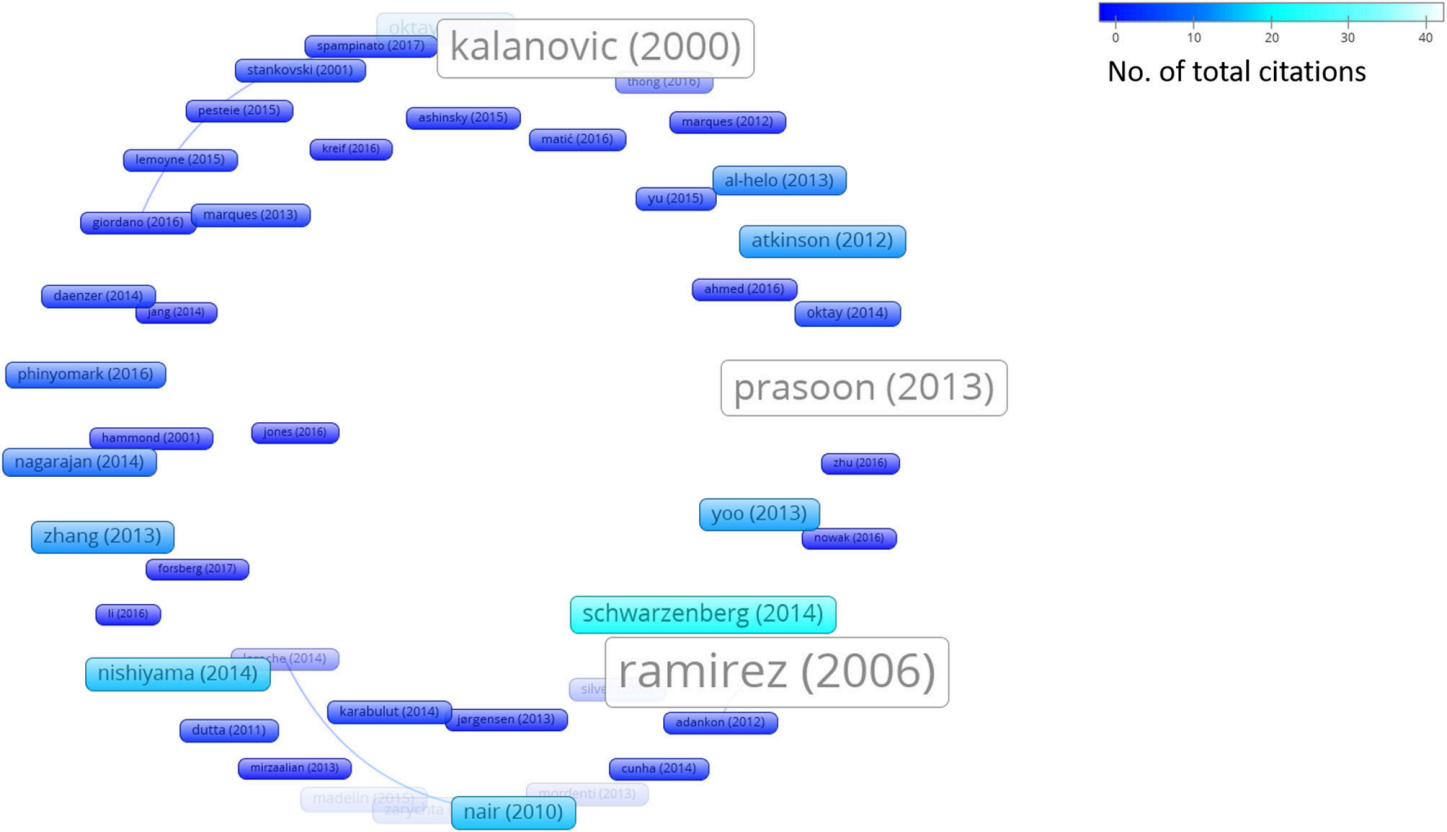
Citation analysis (performed by VOSviewer http://www.vosviewer.com/). Size and brightness levels of the nodes express the number of citations of each paper (the brighter the color, the more citations of that paper). Links connect sources where one paper cites the other one (i.e., the older one).

In addition to this systematic survey summarized in the above charts, we also conducted a graph-based analysis which is related to the academic impact of the research groups contributing to the ML research field and their reputation in this field. To this aim, from among all the papers considered in this survey, we represent both the *bibliographic coupling* in Figure 6 and the *citation analysis* in Figure 7. The bibliographic coupling is defined as the number of common citations (either from within or apart from the set of papers analyzed) within a group of publications; a citation analysis deals with the total number of citations (either inside or outside the set of papers analyzed) that each publication has collected from the subsequent literature production. Thus, Figure 6 presents a corpus of papers where few articles share a relevant number of citations, and the majority of papers share few or no common references. This can be interpreted in terms of a few common inspiring references and, hence, in terms of a related high heterogeneity of the medical and scholarly communities that produced the surveyed articles. Similarly, Figure 7 presents the few papers within our sample that cite each other, thereby corroborating the idea that researchers involved in the application of ML to orthopedic practice do not know (and hence refer to) the similar works of other researchers in the same field. This result should not be considered as an actual sign of community dispersion but rather of the multiplicity of both the ML approaches used thus far and of the orthopedic problems to which ML has been applied. It is reasonable to conjecture that in the next few years, if the trend depicted in Figure 2 is confirmed, the two graphs will grow both in terms of nodes and links, as ML researchers and medical data scientists become more aware of similar works accomplished in their expertise field and more researches will be undertaken in the orthopedic field as well.

**Figure 8:**
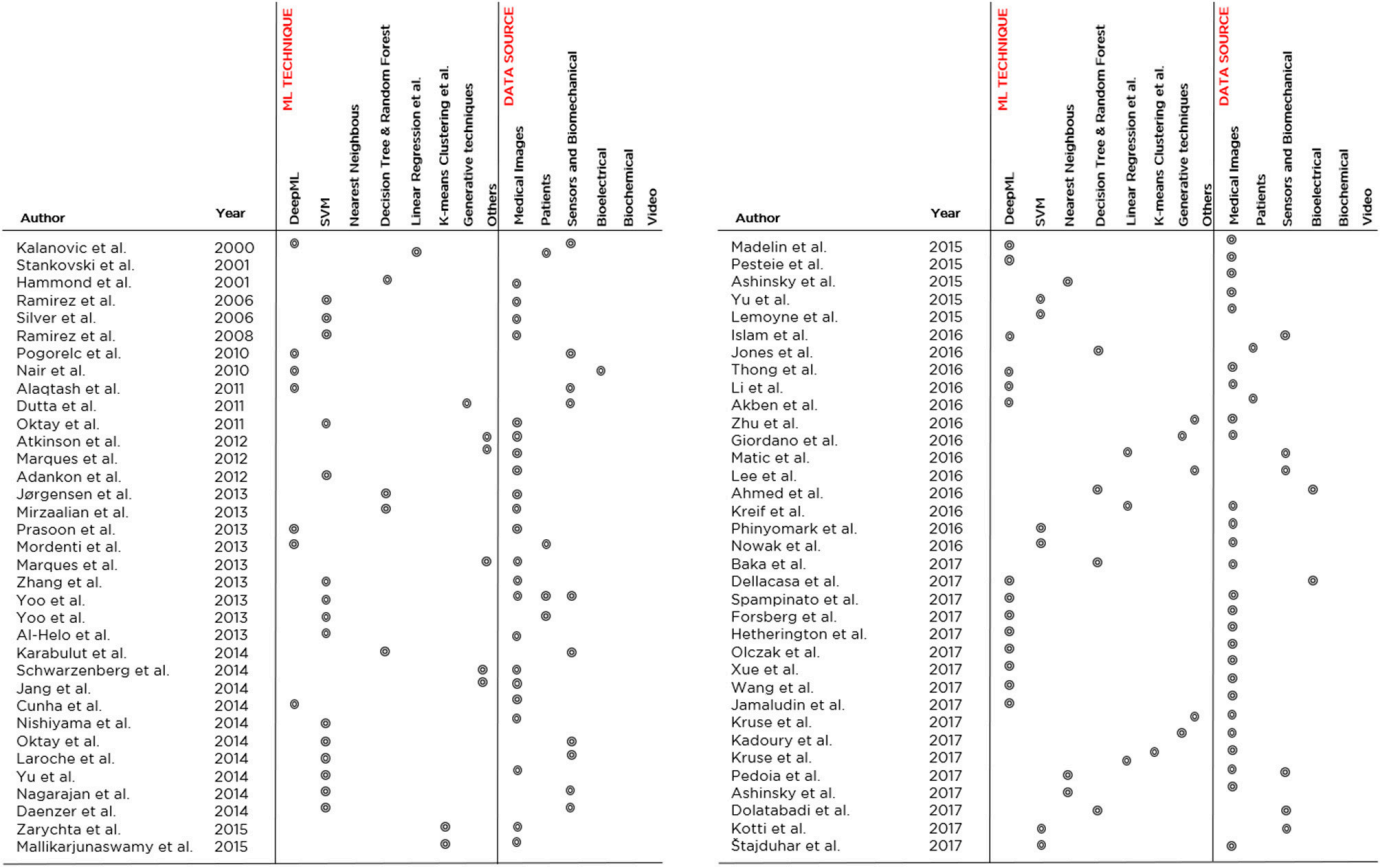
An overview of the 70 papers analyzed in the literature review, indicating the developed ML model, the application domain and the data source typology. The papers are listed in chronological order.

**Figure 9:**
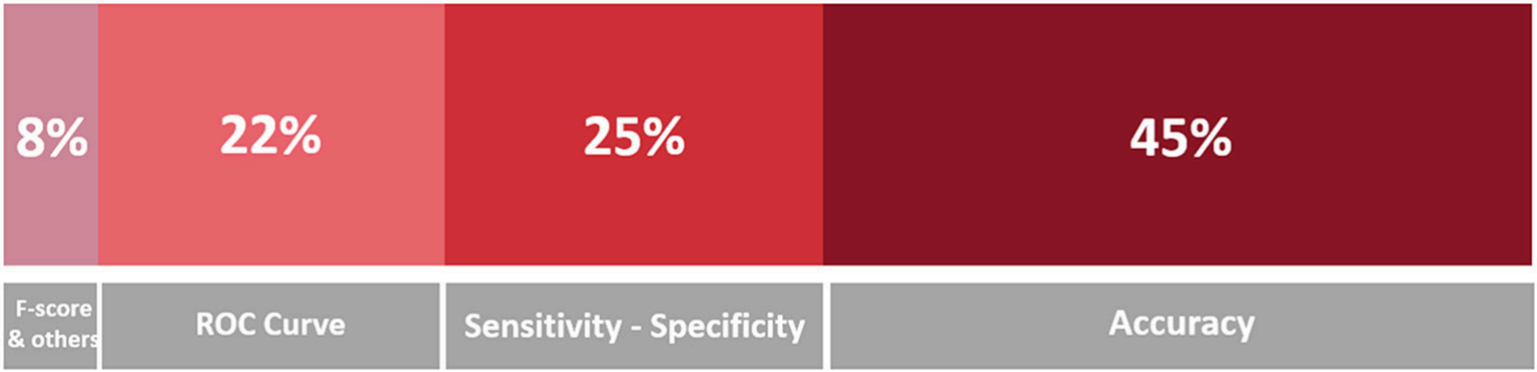
Stacked bar chart representing the proportion of mentions of the evaluation metrics in the surveyed papers. A darker hue indicates a greater number of occurrences, which is also indicated in terms of percentage.

Figure 9 is a stacked bar chart depicting the percentage of the surveyed papers that report in their studies some of the metrics that are recommended in the Standards for Reporting of Diagnostic Accuracy Studies (STARD) statement (Cohen et al., 2016). From this chart, it is evident that the most popular evaluation measure is Accuracy (45% of the publications report accuracy or other measures that can be equated to accuracy, including error rate, mean error rate and success rate), as it was expected; followed by Specificity and Sensitivity (25% of the publications), which are common measures in medical literature; and measures related to the ROC curve (22% of the publications), like the AUC and AUROCor the highest curve value (see the footnote in section 4.1.3 for more details on these measures). Other measures, which are mentioned much less frequently, encompass Precision (AKA true positive rate), Recall (AKA sensitivity), the F-score^7^ (that is the harmonic mean of precision and recall), Inter-rater Agreement (such as the kappa coefficient), and the results of statistical hypothesis tests: all these measures of performance and accuracy model were reported in 6% of the publications, most of the times together with the more common metrics mentioned above. Another metric that is part of “others” in the figure is the Dice Similarity Coefficient, which is used to validate the spatial overlap accuracy in automatic image segmentation tasks.

Examining the best performing model for each class of orthopedic application, on the basis of the highest score of the main evaluation metric considered in the study (discarding the scores below the 85%), we see a varied situation with some results that could seem surprising:indeed, for most application domains, the reported performance is very good (if not almost perfect) like in spine pathology detection (Jamaludin et al., 2017), bone age assessment (Spampinato et al., 2017), prosthesis control (Lemoyne et al., 2015), gait classification' (Pogorelc and Gams, 2010), ostheoartritis detection (Phinyomark et al., 2016) and fracture detection (Atkinson et al., 2012; Al-Helo et al., 2013). In a few cases, like shoulder strength assessment (Silver et al., 2006) and image segmentation Prasoon et al. (2013), there is still room for improvement in the performance. These results seem to corroborate the idea that computers can already outperform human doctors in numerous tasks (Esteva et al., 2017; Brynjolfsson and Mitchell, 2017; Litjens et al., 2017), even in orthopedics (e.g., Jamaludin et al., 2017).

However, in reading of such impressive performances, the careful reader should always consider a number of elements to interpret them appropriately: the ecological setting where the study was accomplished (e.g., either in a laboratory or a real-world setting); how the *ground truth* was created (e.g., how many experts were involved, how consensus labeling was achieved, if any, which is an oft-neglected problem treated in Cabitza et al., 2017a); the volume and heterogeneity (i.e., population representativeness) of the data set used to both train and validate the ML model (assuming that the authors have correctly reported only scores related to the performance of their model on the validation set; this latter one is a different one from the training and test sets); and the “peeking” effect (Diciotti et al., 2013), a kind of data leakage resulting in overfitting and hence low generalisability of the model. In the following and concluding section, we address the delicate task of interpreting ML performance correctly and in light of recent criticism raised against deep learning (Marcus, 2018).

## 5. Conclusion

In this paper, we defined ML as a powerful set of computational tools, as this is the most common meaning of this expression within the medical community. Moreover, we advocated that the assessment of these tools must be strictly related to the main aim for which researchers develop and apply them to medical fields, that is, to support physicians in their main tasks, whose essence is the need to make decisions “in the absence of certitude” (Groopman and Prichard, 2007), and translate these decisions into choices of care for the betterment of patients. This perspective is a pragmatic one and it can contribute to deflating the current rhetoric regarding the coming of age of “artificial intelligence” in medicine (Miller and Brown, 2017), of which ML “rides atop the peak of inflated expectations” (Obermeyer and Emanuel, 2016; Chen et al., 2017), as it urges to ground any expectation on studies conducted in clinical settings and sufficiently powered pilot programs (Steinhubl and Topol, 2018).

Discourses regarding the replacement of human doctors and, similarly extremist stances advocating their intelligence augmentation (Hainc et al., 2017), must not attract the interest of the serious practitioner and must not receive further coverage in the medical literature, where already “too much of the publicized work […in regard to digital health] is characterized by irrational exuberance and excessive hype” (Steinhubl and Topol, 2018). We also concur that these discourses are not useful to twenty-first century medicine (Obermeyer and Lee, 2017), which has to face an increasing demand for its services (also due to population aging) by consumers who have an increasing profile of morbidity and, worse yet, greater and greater expectations on the outcome of their health condition.

Further, the following aspect must also be highlighted: the capability to link a large amount of data and variables together is the main advantage of ML techniques and models with respect to traditional rule-based approaches (Obermeyer and Emanuel, 2016) and conventional regression models (Chen et al., 2017) that have been commonly used in medicine to estimate prognostic scores or suggest diagnostic hypotheses until the recent past. However, while the power of “capturing complex, nonlinear relationships in the data” (Chen et al., 2017) is an unquestionable capability of ML models, this capability is not the entire solution to any inquiry a medical doctor could formulate at the point of care. As also noticed in Obermeyer and Emanuel (2016), ML “algorithms might “overfit” predictions to spurious correlations in the data” and identify accurate predictors that, despite their predictive power, “are not causes.”

Our literature survey for the orthopedic field outlined a still preliminary phase of ML adoption, as only a few studies were available that employed this approach to problems of orthopedic interest with respect to other medical fields (e.g., cardiology, oncology). This notwithstanding, Figure 2 clearly shows an increasing trend of interest for this kind of application in recent years; it is easy to expect that an increasing number of studies and papers reflect the diffusion of user-friendly computing platforms both to adopt conventional techniques (e.g., Weka Smith and Frank, 2016) and develop deep learning models (e.g., TensorFlow Rampasek and Goldenberg, 2016). In other medical fields, such as cardiology (Rajpurkar et al., 2017), opthalmology, and diabetology (Gulshan et al., 2016) and at the intersection between oncology, pathology (Bejnordi et al., 2017) and dermatology (Esteva et al., 2017), ML is obtaining a crescent interest for some specific and circumscribed success, where authors claim to have developed and validated models capable to outperform human specialists (Brynjolfsson and Mitchell, 2017).

Despite these promising results, ML is not different from any other health technology; hence, ML must be assessed and evaluated when applied in real-world settings according to the tenets of Health Technology Assessment—that is, in structured research frameworks whenever possible, like cohort studies and randomized controlled trials. ML must still undergo a number of phase 3 trials before its wide adoption (and related investment) can be recommended, despite the increasing numbers of proponents, both outside and within the medical community. Currently, the application of ML in the orthopedic field is still grounding on phase 2, or even smaller, studies and experimental researches.

In this field, the entrance of private companies^8^, their capability to pour important investments into applied research and to obtain access to large, population-wide data sets is a phenomenon worthy of interest and one that will likely have an impact in upcoming years. This impact is regarding the increased automation of diagnostic and prognostic services and, hence, higher throughput (if not higher accuracy) as well as other promising applications, like a more efficient operating room scheduling, better prosthesis myocontrol, and higher resolution imaging (while keeping or reducing the radiation and exposure time).

These applications and the other ones surveyed in this paper, as well as the high quality performance reported in the studies surveyed, suggest that a new systematic literature review will likely be necessary in the few next years to “stand on the shoulders” of the current one that was aimed at covering the last 20 years.

## Author contributions

All authors listed have made a substantial, direct and intellectual contribution to the work, and approved it for publication.

### Conflict of interest statement

The authors declare that the research was conducted in the absence of any commercial or financial relationships that could be construed as a potential conflict of interest.

## Footnotes

^1^In fact, before the expression ML became popular and almost a buzzword even in medical literature, the same computational techniques were denoted with the more prosaic expressions of “statistical learning” and “data mining,” and did not even have an umbrella term encompassing all of them from the tool box of statistical analysts.

^2^Tree Bagging Bootstrap techniques aggregate weak learners such as Decision Trees, and are based on a bootstrap resampling version of the training data and on majority vote classification (bagging).

^3^A tree in which each node represents a conditional posterior probability computed and propagated to its sub-trees. The top node of the tree represents the overall posterior probability for all its sub-trees.

^4^The Receiver Operating Characteristic (ROC) curve is a popular visual means to convey information regarding the discriminative performance of a model, given specific thresholds and values of sensitivity and specificity. When associated with single values in the 0–1 range, papers may refer to either the Area Under the ROC curve, also known as AUROC (the closer the value to 1, the better the performance), or to the highest value of ROC, that is the ROC curve point that is closer to the leftmost corner of the sensitivity-specificity space. Unfortunately, not many authors specify if they are reporting the AUROC or the highest ROC value and, what is even worse, denote these values simply as ROC or ROC score. In what follows, we will report the ROC scores as these have been reported by the authors of the original contribution.

^5^Overfitting occurs when a model is very good at predicting the training data, that is, in explaining the hidden relationships between the *x* and *y* that are given to the *learner*; however, its performance is poor with new *x* data, that is, its generalizability is low.

^6^Generative methods are probabilistic methods able to generate a statistical model from a set of observed variables, in order to infer new inputs and the relative outputs, rather than just find a model for the existing variable values (the inputs).

^7^The fact that F-scores are seldom reportedm, in approximately 1 paper out of 50, could also reflect that fact that this measure of model performance is not informative in the medical field, where mistakes are not equally bad (false negatives have a greater impact on the patients' health than false positives, which are conversely related to over-treatment and excessive costs), and it is not generally true that true negatives are uninteresting. If an ML model exhibits more false positives than another model, but is associated with fewer false negatives (while having the same true positives), the F-scores of the two models are the same.

^8^For example, Zebra Medical Vision Kashyap (2017) in 2017 began offering a pay-per-use service (SaaS) for compression fractures detection Bar et al. (2017) and bone density evaluation to potentially all health care facilities that could connect and send medical images to its cloud.
